# Fatty Acid Composition at the Base of Aquatic Food Webs Is Influenced by Habitat Type and Watershed Land Use

**DOI:** 10.1371/journal.pone.0070666

**Published:** 2013-08-05

**Authors:** James H. Larson, William B. Richardson, Brent C. Knights, Lynn A. Bartsch, Michelle R. Bartsch, John C. Nelson, Jason A. Veldboom, Jon M. Vallazza

**Affiliations:** Upper Midwest Environmental Sciences Center, United States Geological Survey, La Crosse, Wisconsin, United States of America; Consiglio Nazionale delle Ricerche (CNR), Italy

## Abstract

Spatial variation in food resources strongly influences many aspects of aquatic consumer ecology. Although large-scale controls over spatial variation in many aspects of food resources are well known, others have received little study. Here we investigated variation in the fatty acid (FA) composition of seston and primary consumers within (i.e., among habitats) and among tributary systems of Lake Michigan, USA. FA composition of food is important because all metazoans require certain FAs for proper growth and development that cannot be produced *de novo*, including many polyunsaturated fatty acids (PUFAs). Here we sampled three habitat types (river, rivermouth and nearshore zone) in 11 tributaries of Lake Michigan to assess the amount of FA in seston and primary consumers of seston. We hypothesize that among-system and among-habitat variation in FAs at the base of food webs would be related to algal production, which in turn is influenced by three land cover characteristics: 1) combined agriculture and urban lands (an indication of anthropogenic nutrient inputs that fuel algal production), 2) the proportion of surface waters (an indication of water residence times that allow algal producers to accumulate) and 3) the extent of riparian forested buffers (an indication of stream shading that reduces algal production). Of these three land cover characteristics, only intense land use appeared to strongly related to seston and consumer FA and this effect was only strong in rivermouth and nearshore lake sites. River seston and consumer FA composition was highly variable, but that variation does not appear to be driven by the watershed land cover characteristics investigated here. Whether the spatial variation in FA content at the base of these food webs significantly influences the production of economically important species higher in the food web should be a focus of future research.

## Introduction

Food quality has been shown to strongly influence the behavior, physiology, ecological interactions and evolution of aquatic consumers [1]–[3]. Some aspects of food quality appear to vary greatly among aquatic systems. For example, elemental composition of seston varies among streams in association with variation in watershed land use [4], and such variation influences consumer-driven processes [5], [6]. Less is known about the spatial controls over variation in many other aspects of food quality.

One aspect of food quality thought to be particularly important is lipid (and fatty acid) quantity and quality [7], [8]. FAs function as structural elements (primarily in cell membranes), energy storage molecules and precursors to signaling hormones [7], [9], [10]. Biota vary greatly in their ability to synthesize FA *de novo*. In particular, the enzymes necessary to synthesize long-chained (>20 C atoms), polyunsaturated FAs (PUFAs) are largely restricted to algae [11]. For metazoans, metabolically essential FA (e.g., eicosapentaenoic acid [EPA] and docosahexaenoic acid [DHA]) or their precursors (e.g., α-linolenic acid [ALA]) must be derived from the diet [7], [9], [12]. As a result, the quantity and composition of FA in food resources constitute an important aspect of food quality for aquatic consumers. Limitations in dietary FAs have been shown to limit cladoceran population growth, fish over-winter survival, prey capture by visual-feeding fish, and a large range of other survival-related behaviors and functions [7], [13]–[18].

Large spatial scale controls over variation in the FA composition of either basal resources (such as seston, biofilms or macrophytes) or primary consumers (i.e., species that feed directly on those basal resources) among freshwater systems are largely unknown. Boëchat et al. [19] found that agricultural streams had higher per-mass amounts of essential PUFAs in biofilms than pristine streams, although biofilms were overall less extensive in agricultural streams (i.e., agricultural streams had small amounts of high-quality biofilms). On the other hand, Müller-Navarra et al. [20] found that lake trophic status (indicated by total phosphorus [P]) was negatively correlated to the amount of EPA and DHA in seston (per unit C). Agriculture and other human land uses tend to increase the nitrogen (N) and P available in downstream waters [19]–[21], and these nutrient increases are presumably influencing algal community dynamics (productivity and species composition) in these previous studies [19], [20]. Whether these changes in primary producer FA composition lead to variation among ecosystems in primary consumer FA composition has apparently not been assessed.

**Figure 1:**
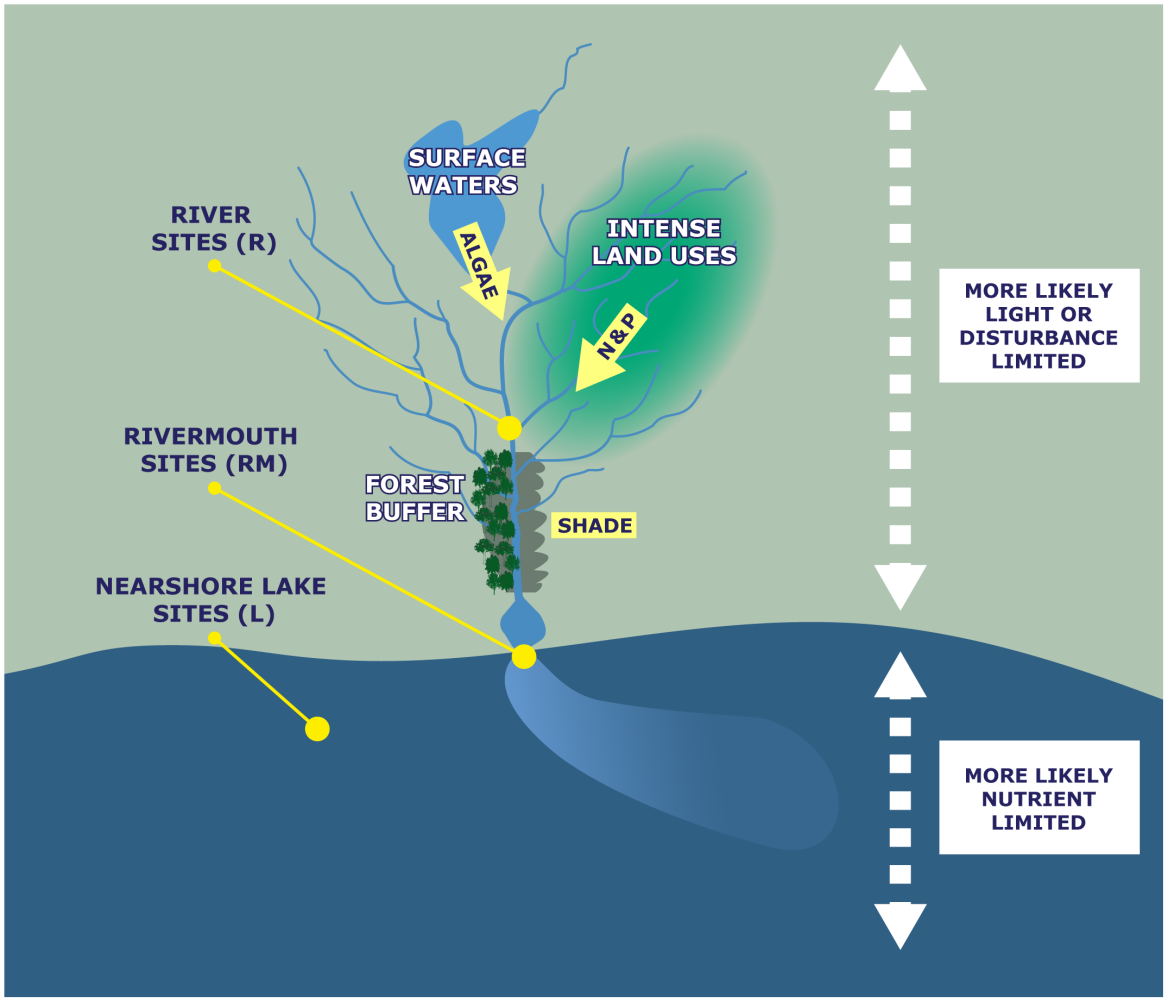
Conceptual figure showing predictions and study design in the context of a hypothetical tributary system. Our underlying prediction is that watershed land cover significantly influences fatty acid (FA) content of seston and consumers via effects on algal production and composition. The land cover properties investigated here are highlighted in white font (surface waters, intense land uses, and forest buffers) with their presumed mechanistic relationship to algal dynamics in yellow boxes or arrows (algal export, nitrogen [N] and phosphorus [P] loading and stream shading). Habitat types sampled in this study are also shown (river, rivermouth and nearshore lake sites).

The objective of this study was to 1) assess variation among Lake Michigan tributary systems in the quantity and quality of FAs at the base of food webs and 2) determine if watershed land cover influences this among-system variation. Our primary hypothesis is that watershed land cover influences the FA composition of seston and primary consumers via variation in algal production and composition among ecosystems (Figure 1). Specifically, we predict three watershed characteristics might influence algal growth and composition. First, intense human land use (i.e., agriculture and urban development) increases nutrient loading, thus potentially increasing algal production [19]–[21]. Secondly, forested riparian buffers both shade streams and intercept nutrients from upstream developed areas, potentially reducing algal production [22], [23]. Finally, greater numbers of lakes and impoundments (i.e., surface waters) in the watershed, will result in greater water retention times and anticipated algal production available for export downstream [24]. Thus we predict that these three watershed characteristics (i.e., intense land use, forested buffers and surface water) will have significant effects (positive, negative and positive effects, respectively) on PUFAs and other FA in seston and consumers tributary systems (Figure 1). To test these predictions, FAs were measured in seston and primary consumers from 11 tributary systems of Lake Michigan in three habitat types (rivers, rivermouths and the nearshore zone adjacent to the rivermouth).

The habitat types sampled here differ in the underlying controls over ecosystem processes (e.g., productivity) and thus potentially connections to the watershed [25], [26]. For example, rivers are often highly shaded with productivity limited by incident light [27] and thus rivers often react differently than lakes to increases in nutrients [28] (Figure 1). The rivermouths sampled here probably are not light-limited and would probably be more lake-like in response to nutrients, although estuaries (marine analogues to the rivermouths sampled here) show complex eutrophication dynamics [29]. The tributary watersheds of Lake Michigan are ideal for this type of study because land cover varies among them from intense row crop to primarily forest covered [30].

## Materials and Methods

### Ethics Statement

No permits were required for the sample collection described herein. All of the sites sampled here are publicly accessible and no threatened or endangered species were collected as a part of this study.

### Study Sites

**Figure 2:**
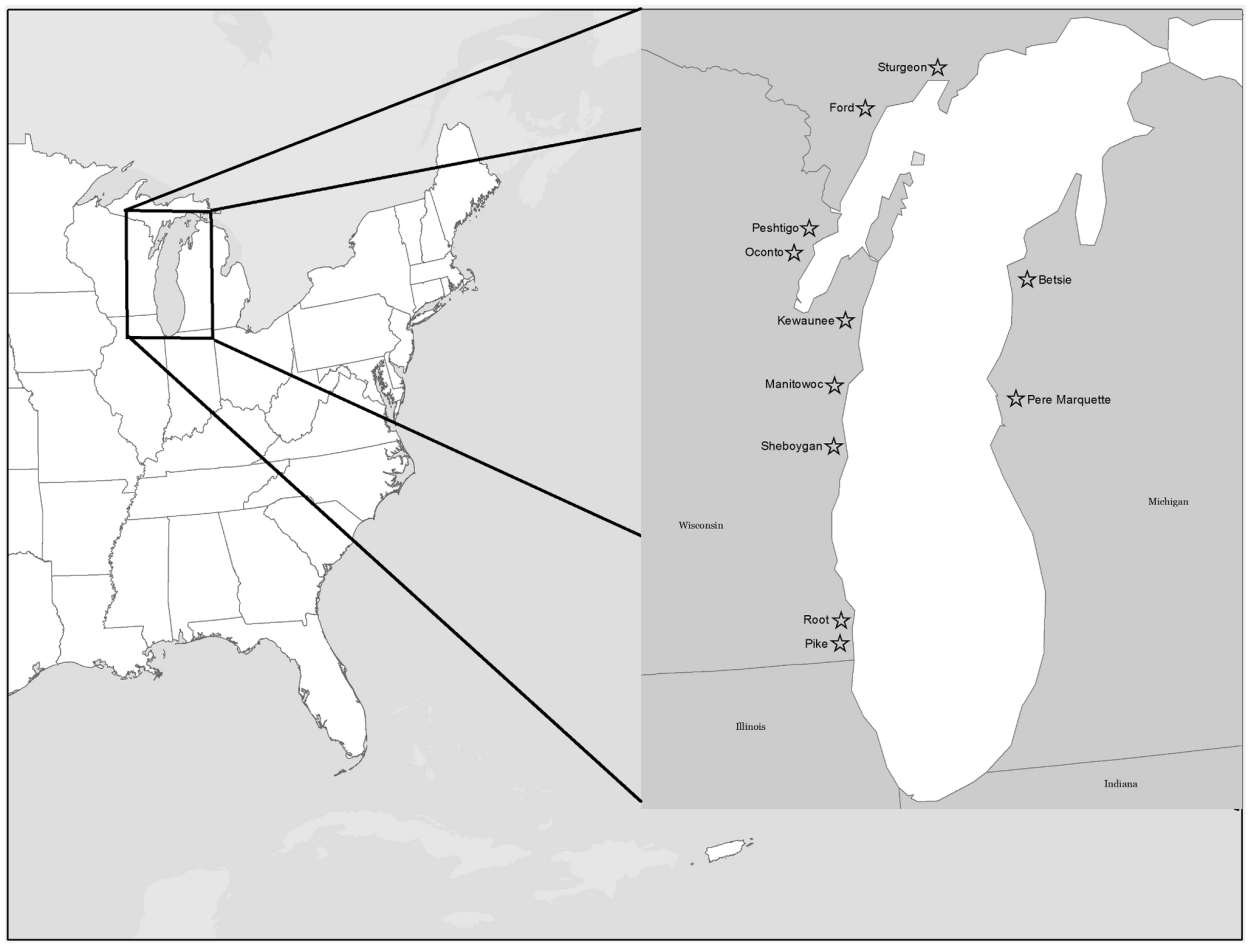
Location of rivermouth systems sampled during this study. More detailed geographic information is available in Appendix S1.

Eleven tributary systems of Lake Michigan were sampled during September and October of 2010 that varied in catchment land use (Figure 2, Table S1, Appendix S1). One site at each of three habitat types was sampled for seston in each tributary system including 1) at the most downstream USGS gage station in the river (river; R), 2) at the confluence with Lake Michigan (rivermouth; RM) and 3) about 200 meters into Lake Michigan (nearshore zone of lake; L; see Figure 1). Only minimal surface water inputs, as assessed with aerial photography, occurred between R and RM sites. At L sites, water samples were taken outside of the river plume as determined by conductivity (Lake Michigan conductivity was typically distinct from river water; see data in [26])and visual cues (i.e., plumes often were visibly stained or turbid). The Betsie River had no gage station; therefore, a river site outside of any apparent seiche influence (∼20 km upstream of the rivermouth) was sampled.

### Seston

At each location, surface water (10–20 cm depth) was collected in plastic bottles, placed into coolers with ice and returned to a lab for filtering (within 6 hours). Seston was collected by pre-filtering water through a 35 µm Nitex® mesh (to exclude particles above 35 µm) and then seston <35 µm was retained on 6 glass fiber filters (Pall A/E; 0.9 µm, 47-mm diameter), two of which had been pre-weighed for suspended solids analysis. Two filters were stored in cryovials in liquid nitrogen and returned to the Upper Midwest Environmental Sciences Center (UMESC) for lipid and FA analysis. Two filters were frozen and returned to UMESC for chlorophyll *a* analysis. Chlorophyll *a* was determined spectrophotometrically [31]. The two pre-weighed filters were frozen, returned to UMESC, dried at 103–105°C and reweighed to determine total suspended solids (TSS).

### Caddisflies

At nine of the R sites, filter-feeding caddisflies (Family Hydropyschidae) were collected from available substrate, usually rocks, but in some cases woody debris. Between 5 and 10 individuals were combined into a single sample and stored in cryovials in liquid nitrogen until they were returned to UMESC in La Crosse, WI for analysis of lipids and fatty acid. Several individuals were collected as voucher specimens (stored in 95% ethanol) and later identified to genus [32]. Hydropyschid caddisflies could not be found at the Ford or Sturgeon R sites. At some sites, duplicate samples were taken (i.e., a second set of 5–10 individuals) and treated identically.

### Dreissenids

At RM sites, dreissenid mussels were collected from interior breakwalls or rocky substrate. Dreissenid mussels for L sites were collected from exterior or other sites along the shoreline within about 1-km of L seston collection sites. Between 3 and 5 individuals (∼2–3 cm in length) were combined into a single sample. All were collected at less than 1 m of depth. Mussel soft tissue (without byssal threads) was removed from shells and stored in cyrovials in liquid nitrogen until they could be returned to the lab for processing. All individuals appeared morphologically to be *Dreissena polymorpha*, although cryptic occurrence of *D. quagga* cannot be ruled out [33]. The dreissenid mussel sample at the Betsie L site was lost after collection. At RM sites for the Sturgeon and Ford, no dreissenid mussels could be found. At some sites, duplicate samples were taken (i.e., a second set of 3–5 individuals) and treated identically.

### Lipid Analysis

At UMESC, samples were freeze dried in a Virtis freeze dryer (SP Scientific, Gardiner, NY) and stored at −80°C until analysis. Tissues were homogenized by grinding in liquid nitrogen in preparation for FA analysis. Methods for extraction and analysis of FA followed Hebert et al. [34] with an internal standard (5-α-cholestane, Sigma-Aldrich C8003) added to each sample prior to extraction to estimate percent recovery and a free fatty acid (17∶1ω7) was added prior to methylation to estimate methylation efficiency. The methylated lipid extract was analyzed by a gas chromatograph (Agilent Model 7890, Wilmington, DE) using a flame ionization detector, with a Supelco 2560 capillary column (100 m, 0.25 mm inner diameter and a 0.2 µm film thickness). On about 4.5% of the samples we ran procedural replicates (i.e., splitting the sample in half and separately extracting and methylating each half). These replicates showed small variation (<2% variation in total FAs). Procedural replicates were averaged for a single sample. For sites with duplicates, the average of the duplicates is reported as the site value. Raw data for all FA samples (including replicates and duplicates) is included in the S2.

A total of 42 fatty-acid methyl esters were identified by comparison of their retention times with known standards (see Table S2). Here the nomenclature *A*:*B*ω*C* is used, where *A* is the number of carbon atoms, *B* is the number of double bonds and *C* is the position of the first double-bond relative to the terminal (ω) methyl carbon atom. Structurally, fatty acids (FAs) vary in the length of the carbon (C) chain, the number of unsaturated C-C bonds (i.e., a double bond between adjacent C atoms) and the position of those unsaturated C-C bonds [35]. Of the 42 fatty acids analyzed, five polyunsaturated fatty acids (PUFAs) thought to be metabolically essential fatty acids (EFAs) or precursors for those EFAs for most metazoan consumers were individually used as indications of food quality [9], [12]: 18∶2ω6, linoleic acid (LIN), 18∶3ω3 α-linolenic acid (ALA), 20∶4ω6 arachidonic acid (ARA), 20∶5ω3 eicosapentaneoic acid (EPA) and 22∶6ω3 docosahexaenoic acid (DHA). In addition, five aggregations of FA were calculated: the sum of all ω3 FAs, the sum of all ω6 FAs, the sum of all PUFAs, the sum of all monounsaturated FA (MUFAs) and the sum of all fatty acids (∑FA; see complete list in Appendix S3). The sum of ω3 and ω6 FAs were used to calculate ω3:ω6 ratios. For seston, these 10 FA variables were expressed in two ways: per liter of water filtered (*seston FA quantity*) and per mg total suspended solids (*seston FA quality*). Consumer FAs were reported per mg dry mass.

### Watershed Land Use

Land use metrics for the 11 tributary watersheds were based on watershed boundaries from the USGS Watershed Boundary Dataset (WBD) and user-defined boundaries created from 10-meter DEMs from the USGS National Elevation Dataset (NED). The entire watershed basin properties (above the RM sampling location) were calculated based on the WBD and the properties above the R locations were based on those created from 10-meter NED using Pour Point in ArcGIS 10.0 [36]. After the watershed was delineated, summaries of land use based on the 2001 National Land Cover Database were determined [37]. The open-water land-use type represented lakes and other surface waters in the watershed (Surface water). For most sites, surface water calculated at the R and RM locations was similar, indicating the distribution of surface water was not focused at the rivermouth itself. However, for the Betsie River, just under half of the entire watershed’s surface water (at the RM) was located below the R site in a drowned rivermouth lake. An intense disturbance category (Intense land use) was created from a combination of Cultivated Crops and all of the Developed classes (‘Developed, Open Space’, ‘Developed, Low Intensity’, ‘Developed, Medium Intensity’ and ‘Developed, High Intensity’). Total hectares in the entire watershed and in these individual categories were used to calculate the proportion of the watershed that was either surface water or intense land use.

The riparian buffer was created by drawing a 50 m buffer around all of the streams within the watershed identified in the National Hydrography Dataset. Within that buffer, summaries of land cover based on the 2001 National Land Cover Database were created [37]. From these, the Deciduous Forest, Evergreen Forest and Mixed Forest categories were combined into a single category (forested buffer) and the hectares of this category were divided by the total hectares of the buffer to estimate the proportion of the buffer that was forested.

### Statistical Analysis

Statistical analyses were conducted in R Version 2.11.1 [38]. Pearson correlation coefficients were calculated for seston and consumer FA metrics using the “cor()” function. Mean values and 95% credible intervals around the mean for seston and consumer FAs at R, RM and L sites were made using the approach described in McCarthy, pp 66–67 [39] (see example code in Appendix S3). Statistical comparisons between R, RM and L sites in mean FA values were made by assessing overlap between 95% credible intervals. In this approach, a ‘significant’ difference between habitat types is inferred when 95% credible intervals do not overlap.

Prior to estimating relationships between FA properties of seston and consumers and land cover, individual values for each predictor and response variable were standardized by subtracting the mean and dividing by the standard deviation (creating variables where the mean is zero and the standard deviation is 1; [40]). Land cover and FA properties were standardized within habitat type (R, RM, L). Simple linear regression models between standardized land cover and response variables were created using the BRugs package in R, following the approach described in McCarthy ([39]; see example code in Appendix S3). This is a Bayesian regression approach (using uninformative priors) that estimates the slope with 95% credible intervals. A statistically “significant” slope is defined here as one where this credible interval does not overlap zero [39], [41]. Since these are standardized slopes (sometimes referred to as beta slopes), the magnitude of the slope can be compared among variables that differ in units [40].

## Results

### Variation in Seston and Consumer FAs Among Tributary Systems and Habitat Types

Variation in individual and aggregate FA metrics was strongly correlated to the ∑FA in both seston and consumers (Table S3). Exceptions include EPA content of caddisflies and ARA content in dreissenid mussels (Table S3). Therefore, for seston the following analyses focus on ∑FA, but the trends are similar for PUFAs, MUFAs and the individual fatty acids measured here (those analyses are presented in Appendix S2). Similarly, the presented analyses for consumers focus on ∑FA, ARA and EPA, with other FA metrics following similar trends as ∑FA (Table S3).

**Figure 3:**
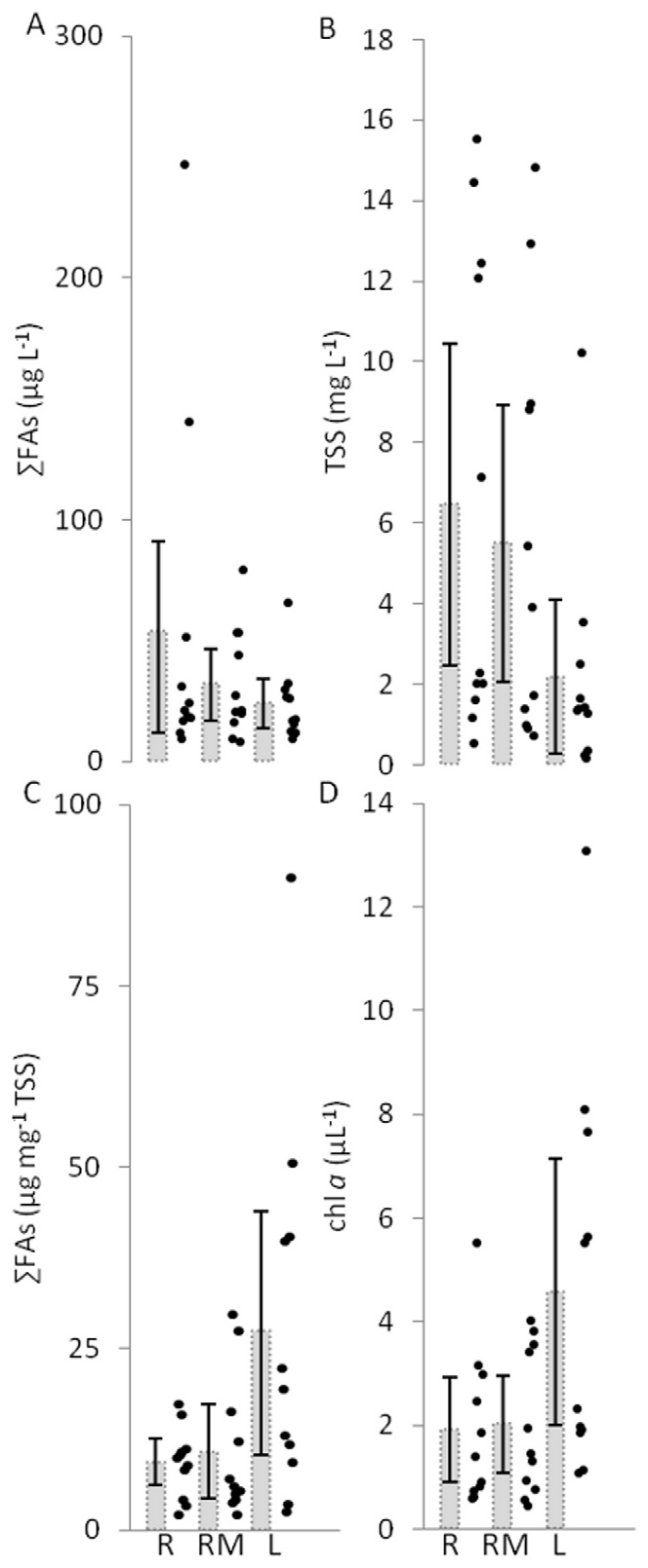
Total fatty acid (∑FA) quantity and as a proportion of the total seston, total suspended solids (TSS) and chlorophyll *a* (chl *a*) in rivers (R), rivermouths (RM) and the nearshore of Lake Michigan (L). Raw data (points) and averages (with 95% credible intervals) are shown here. Although not presented, the trends in ∑FA in seston largely mirror trends in other seston FAs measured here (see Table S3 and Appendix S2). Statistical significance is inferred by the lack of overlap between credible intervals (no statistically significant differences occur here).

There were no statistically significant differences in the average seston FA quality or quantity, TSS or chlorophyll *a* among R, RM and L sites (Figure 3, Table 1). Variability in seston FA quantity (µg per L of water) was higher among R than L sites (Figure 3A), and variability in TSS was high in all habitat types (Figure 3B). Variability in seston FA quality (µg per mg TSS) was higher among L sites than R or RM sites (Figure 3C), as was also the case for chlorophyll *a* (Figure 3D).

**Table 1:** Mean and 95% credible intervals of seston and consumer response variables sampled at river (R), rivermouth (RM) and nearshore lake (L) sites (number of sites indicated in *[brackets]*).

Variable	Units	Media	R*	RM*	L*
∑FA	µg L^−1^	Seston	34.00_(−7.74 to 71.16)_ [*11*]	30.36_(15.32 to 44.49)_ [*11*]	23.41_(12.78 to 33.66)_ [*11*]
PUFA			18.75_(−5.22 to 41)_	10.41_(5.01 to 15.68)_	8.93_(4.06 to 13.69)_
MUFA			9.02_(1.2 to 16.79)_	5.98_(2.93 to 9.04)_	4.81_(2.57 to 7.05)_
EPA			7.65_(−2.51 to 17.74)_	3.12_(1.27 to 4.99)_	1.61_(0.75 to 2.47)_
DHA			1.79_(−0.36 to 3.91)_	1.1_(0.5 to 1.693)_	0.82_(0.44 to 1.197)_
ARA			0.51_(0.02 to 1.00)_	0.38_(0.11 to 0.65)_	0.39_(0.19 to 0.60)_
ALA			4.45_(−0.7 to 9.616)_	1.65_(0.9 to 2.41)_	2.23_(0.74 to 3.73)_
LIN			1.59_(−0.06 to 3.23)_	0.75_(0.39 to 1.11)_	1.32_(0.4 to 2.23)_
∑FA	µg mg^−1^ TSS		9.33_(6.13 to 12.57)_	10.71_(4.21 to 17.24)_	25.6_(8.33 to 42.01)_
PUFA			2.87_(1.43 to 4.31)_	3.66_(0.89 to 6.42)_	10.86_(3.58 to 18.07)_
MUFA			1.72_(0.99 to 2.44)_	1.97_(0.78 to 3.15)_	5.16_(2.04 to 8.25)_
EPA			0.92_(0.29 to 1.54)_	0.99_(0.22 to 1.75)_	1.72_(0.72 to 2.72)_
DHA			0.23_(0.1 to 0.37)_	0.51_(−0.12 to 1.13)_	1.15_(0.37 to 1.94)_
ARA			0.08_(0.04 to 0.12)_	0.1_(0.05 to 0.16)_	0.47_(0.17 to 0.77)_
ALA			0.57_(0.25 to 0.9)_	0.65_(0.1 to 1.21)_	2.77_(0.77 to 4.77)_
LIN			0.27_(0.13 to 0.41)_	0.3_(0.09 to 0.5)_	1.74_(0.45 to 3.04)_
ω3:ω6	none		1.68_(1.24 to 2.12)_	1.55_(1.18 to 1.91)_	1.22_(1.07 to 1.37)_
Chl *a*	µg L^−1^		1.91_(0.89 to 2.937)_	2.02_(1.09 to 2.966)_	4.56_(2.00 to 7.129)_
TSS	µg L^−1^		6.45_(2.45 to 10.42)_	5.49_(2.06 to 8.907)_	2.19_(0.28 to 4.083)_
∑FA	µg mg^−1^ DW	Consumer	72.00_(39.27 to 95.23)_ [*9*]	53.85_(14.21 to 84.3)_ [*9*]	40.93_(30.56 to 50.28)_ [*10*]
PUFA			31.98_(24.58 to 38.93)_	30.28_(17.22 to 42.24)_	21.41_(16.68 to 26.06)_
MUFA			24.04_(15.84 to 31.89)_	17.8_(5.27 to 29.76)_	7.927_(5.86 to 9.99)_
EPA			13.68_(9.99 to 17.33)_	9.89_(4.34 to 15.38)_	5.27^(3.47 to 7.05)^
DHA			0.7168_(0.38 to 1.05)_	4.293_(3.05 to 5.54)_	3.007_(2.47 to 3.54)_
ARA			1.67_(1.3 to 2.05)_	2.08_(1.81 to 2.36)_	2.54_(2.24 to 2.85)_
ALA			9.15_(5.3 to 12.94)_	4.56_(1.11 to 7.99)_	1.98_(1.15 to 2.81)_
LIN			3.76_(2.45 to 5.08)_	1.90_(0.97 to 2.85)_	1.65_(1.00 to 2.31)_
ω3:ω6	none		3.23_(2.25 to 4.22)_	2.06_(1.69 to 2.43)_	1.45_(1.05 to 1.85)_


∑FA - total fatty acids; Chl *a* - chlorophyll *a*; TSS - total suspended solids <35 µm), PUFA - polyunsaturated FA; MUFA - monounsaturated FA; LIN - 18∶2ω6, linoleic acid; ALA - 18∶3ω3 α-linolenic acid; ARA - 20∶4ω6, arachidonic acid; EPA - 20∶5ω3, eicosapentaneoic acid; DHA - 22∶6ω3, docosahexaenoic acid.

* R consumers sampled were caddisflies (Family Hydropyschidae). RM and L consumers sampled were dreissenid mussels (presumably *Dreissenna polymorpha*).

**Figure 4:**
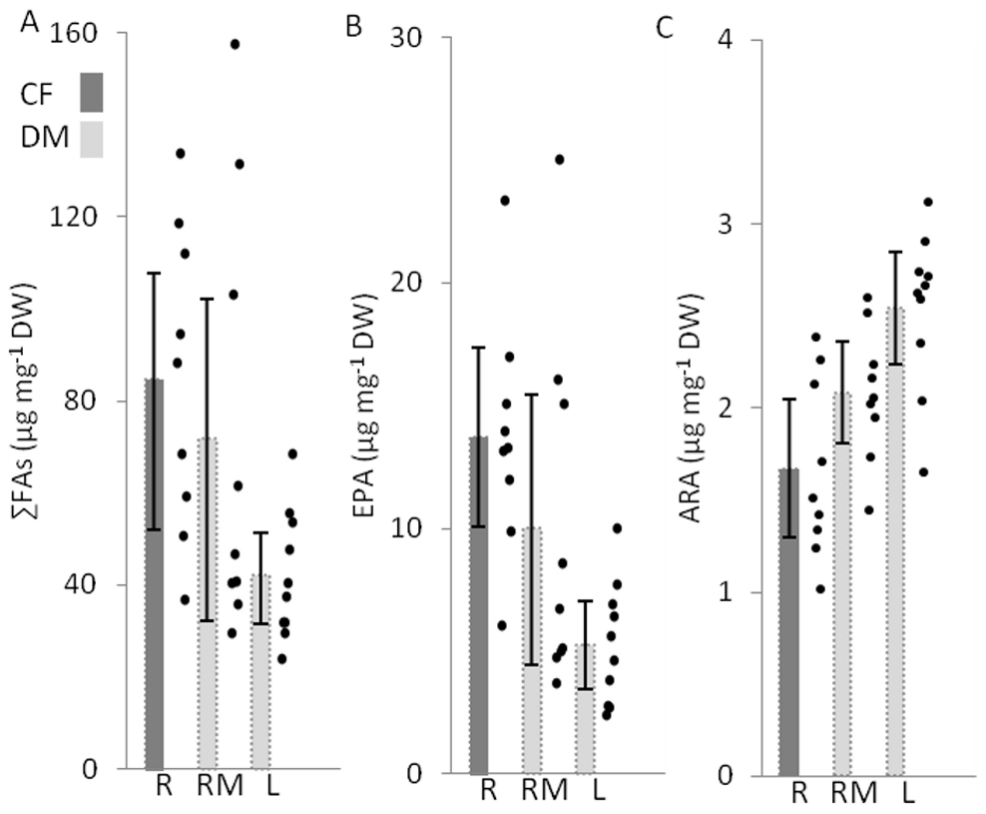
Total fatty acid (∑FA), EPA and ARA in river (R) caddisflies, and dreissenid mussels collected in rivermouths (RM) and the nearshore of Lake Michigan (L). Raw data (points) and averages (with 95% credible intervals) are shown here. Although not presented, the trends in ∑FA in seston largely mirror trends in PUFAs, MUFAs, DHA, ALA and LIN (see Table S3 and Appendix S2). Statistical significance is inferred by the lack of overlap between credible intervals (no statistically significant differences occur here). To highlight differences in taxa, the bar representing caddisflies has been shaded darker.

Consumer FAs cannot be compared as easily among habitats because no common consumer was found. Previous work in the Mississippi River suggests that comparisons between caddisflies and dreissenid mussels might not be appropriate (Knights unpub. data). The only within-taxa, among habitat comparisons are between dreissenid mussels at the RM and L sites. As with the seston, there were no statistically significant differences between RM and L dreissenid mussel FAs (Figure 4, Table 1). RM dreissenids did show much higher variability in FA content than nearshore dreissenids due to the presence of a few sites with extremely high values.

### Is Among Tributary System Variation in Seston FA Influenced by Watershed Land Cover?

**Figure 5:**
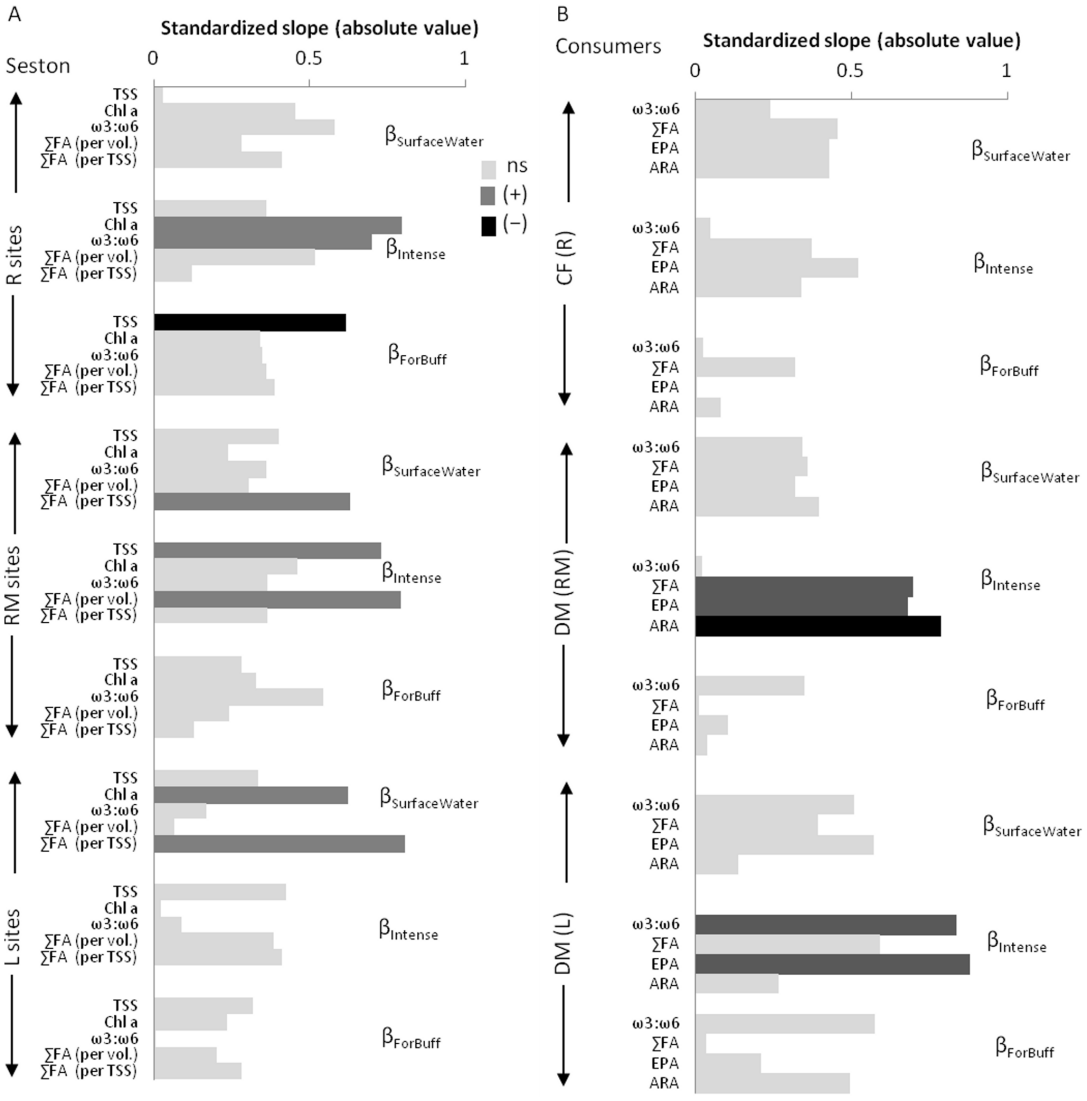
Standardized slopes (β) between land cover characteristics and fatty acid (FA) properties. A) Relationships between land cover and seston TSS, chlorophyll *a* and FA properties at river (R), rivermouth (RM) and nearshore lake Michigan sites (L). B) Relationships between land cover and consumer FA properties at R, RM and L sites. Other measured FA metrics not presented here vary in the same manner as total fatty acids (∑FA; Table S3). Slopes where 95% credible intervals overlapped zero are colored light grey and indicate non-significant (ns) relationships. Statistically significant positive (+) relationships are shaded dark grey, while statistically significant negative (−) relationships are shaded black.

**Figure 6:**
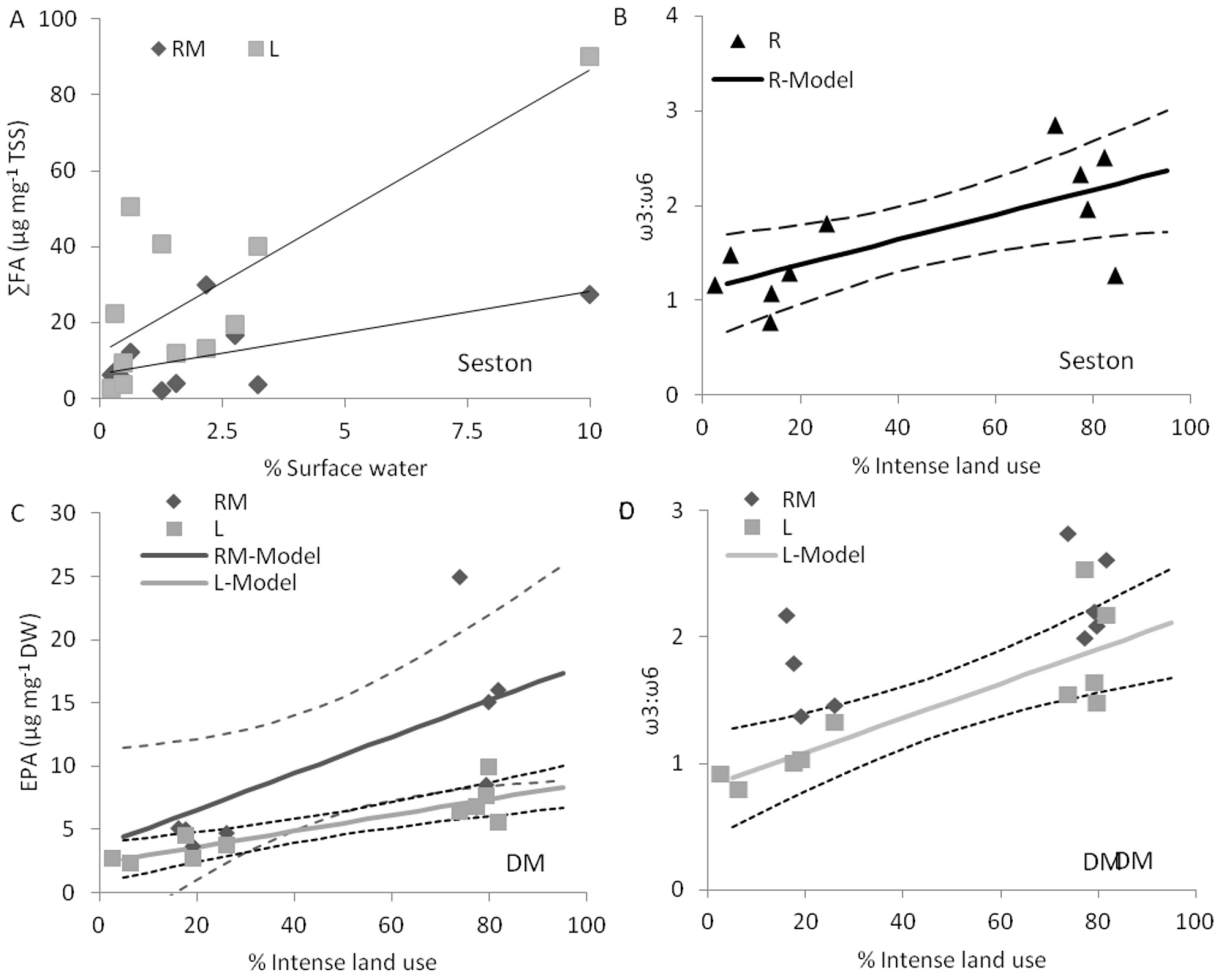
Relationships between selected FA properties and land cover characteristics. A) The percent of the watershed that is open water versus the total fatty acids (∑FA) in seston. Solid and dashed lines show the model at rivermouth (RM) and nearshore lake (L) sites, respectively. This relationship is driven by a single outlier, casting doubt on its usefulness, so no credible interval has been estimated. The proportion of the watershed that is intense land use and the B) seston ω3:ω6 at river (R) sites, C) dreissenid mussel (DM) EPA at rivermouth (RM) and nearshore zone (L) sites and D) DM ω3:ω6 in L sites. For statistically significant relationships in B-D, the solid lines show the model and dashed lines of the same color indicate 95% credible intervals of the estimated model.

Regressions between standardized seston FA and land cover data revealed some significant relationships (Figure 5A). Intense land use was positively related to seston FA quantity in RM sites and the ω3:ω6 ratio in R sites (Figure 5A, Figure 6B). Percent surface water was positively related to seston FA quality and chlorophyll concentration in the RM and L sites (Figure 5A). However, a single outlier appears to be driving these two relations (the Betsie system; Figure 6A). The seston FA quantity and quality were unrelated to the proportion of forested buffer, although TSS was negatively related to forest buffer (Figure 5A). Overall, two of the three land cover characteristics investigated here seem to have weak relationships with seston FAs (surface waters and forested buffer).

### Is Among Tributary System Variation in Consumer FA Influenced by Watershed Land Cover?

Regressions between land cover characteristics and caddisfly FAs were weak (Figure 5B). Dreissenid mussel FA metrics were strongly related to intense land cover at RM and L sites (Figure 5B). In dreissenid mussels at RM sites, increased ∑FA and EPA and decreased ARA was related to increased intense land use. Additionally, EPA and ω3:ω6 increased in dreissenid mussels at L sites with increasing intense land use in watersheds (Figure 5B, Figure 6C-D).

## Discussion

### Do Watershed Characteristics Influence FA Composition at the Base of Aquatic Food Webs?

We predicted that land cover controls over algal production might lead to relationships between land cover and FA content in seston and primary consumers. In particular, we predicted that FAs would be positively related to the proportion of intense land use and surface waters in the watershed, and negatively related to forested buffer (Figure 1). Of the three watershed characteristics predicted to be important, only intense land use appeared to be strongly related to the FA quantity in seston or consumers. Even for intense land use, relationships with FA metrics were only strong in two habitat types (rivermouth and lake). Intense land use might be more important in the rivermouth and lake (compared to the river) because of underlying differences in the controls on algal production in these different habitat types. The light regime of rivers is both highly variable and of immense importance to algal production and thus algal FA composition [42]–[44]. Lotic waters also carry high levels of suspended detritus as part of their seston and have short flushing times that reduce the development and accumulation of phytoplankton compared to more lentic systems [4], [45], [46]. Increases in anthropogenic nutrients therefore may not be directly related to variation in riverine algal production simply because so many other factors must also be favorable (e.g., position of a site within a pool-riffle sequence, frequency of periphyton sloughing, light penetration) [28].

A positive relationship between FAs and surface waters was also apparent in this data, but our confidence in this relationship is weak because of an influential outlier with a high proportion of surface waters (i.e., the Betsie River with 9%). If this relationship is true, only a small proportion of rivermouths in the Great Lakes watershed seem likely to be strongly influenced by this effect. Of the 2,100+ rivermouths on the U.S. side of the Great Lakes, only 55 have ≥9% of their watershed in surface waters (Nelson, J.C. pers. comm.). Additional data on tributary systems with a high proportion of surface water is needed to be more confident in this relationship.

Although the seston samples taken in this study are a snapshot, the consistency between mussel (which accumulate over longer time periods) and seston results at the rivermouth sites suggest the role of intense land use is important. This finding supports our underlying hypothesis that land cover characteristics might be influential in determining algal FA production. However, this support is limited (to rivermouths) and the controls over the variation observed at riverine sites remain largely unknown. This exploratory study did not take into account many factors that might be important for river habitats. For example, reach-scale characteristics (the location within the pool-riffle sequence), antecedent flow conditions (the recent occurrence of large discharge events that might suspend sloughed periphyton) and the spatial sequence of land cover features (whether impoundments occur in headwaters or lower reaches) might all be more important in determining algal dynamics than variation in watershed land cover.

### Spatial Variation Among Tributary Systems and Comparisons to Other Studies

Although FAs are believed to be an extremely important aspect of food quality, there have been few studies that have explicitly investigated among-system variation in the composition of FAs in basal resources [19], [20]. Most studies have focused more on proximate causes of that variation [20]. Direct comparisons between the seston FAs measured here and the seston FAs measured in other studies are difficult due to differences in both sampling approach (e.g., Müller-Navarra et al. deliberately sampled lakes in locations that would have high quantities of algae [20]) and the method of expressing the data (e.g., some authors use proportions [44], others report FA per unit carbon [20] and others report per mass [47]). Müller-Navarra et al. [20] observed almost 2 orders of magnitude variation in EPA and DHA per seston carbon across 13 lakes (although sampled lakes were deliberately selected to span a trophic gradient). Kainz et al. [47], sampling 6 coastal lakes on Vancouver Island, found considerably less variation in seston FA per dry weight, although still a four-fold difference between the highest and lowest levels of DHA and EPA among lakes. Boechat et al. [19], looking at microbial mats in streams, found total FAs per mass varied by just less than 1 order of magnitude. In this study, seston FAs varied between 1 and 2 orders of magnitude among tributary systems, depending on the metric and habitat type. This degree of variation in FA content would lead to variation in the growth and reproduction of consumers [20].

To our knowledge, no studies in freshwaters have directly investigated the controls over spatial variation in primary consumer FA content. In this study, total FAs in a single consumer species ranged 3–5 fold across nine rivermouths. This variation could have significant implications for growth, health and reproduction of predators feeding on these consumers [7], [14]. For example, the open waters of Lake Michigan have become increasingly oligotrophic over the past ten years as a result of rapidly increasing dreissenid mussel populations and efforts to reduce nutrient loading [48], [49]. Because Great Lakes coastal wetlands, rivermouths and river plumes often have more available nutrients than the lake, these non-lake habitats may be increasingly important as centers of primary and secondary production [50]–[52]. The results of this study suggest that those rivermouths will vary considerably in their ability to support healthy populations at higher trophic levels.

This is the second study to document increased levels of FAs at the base of aquatic food webs in response to increasing anthropogenic development [19], although it is the first to document these effects extending to consumers. Presumably, the gradient in intense land use observed here corresponds to a gradient in P loading [53], [54], which we had predicted would increase algal production and thus increase FAs. This interpretation is at odds with the results of Müller-Navarra et al. [20], who found increasing P in lakes lead to decreasing seston EPA and DHA (via changing algal species composition). However, the connection between P loading and eutrophication is less direct in riverine and estuarine ecosystems than it is in lakes [28], [29], and P loading often has a subsidy-stress relationship with important ecosystem properties [55]. Although the gradient in land use sampled here is extensive (from <5% to >80% of the watershed) and probably does correspond to a gradient in nutrient loading, perhaps nutrients are never high enough to cause the shifts in algal community composition that Müller-Navarra et al. [20] observed.

Future work is needed to identify both the portions of the food web that are responding to these gradients in land use, and the effect these changes in FAs near the bottom of the food web might have on economically important species in higher trophic levels. In this study, the consumers sampled were primarily filter-feeders (caddisflies do also opportunistically consume other food resources): Whether other functional feeding groups and taxa would respond similarly is unknown. Indeed, a variety of other factors might influence FA composition at these low trophic levels: for example temporal changes in producer composition, consumer ontogenic changes and weather conditions. If FAs are an important control over secondary production in aquatic food webs, then exploring these real-world controls should be pursued in future research efforts. Future work is also needed to more explicitly link riverine and rivermouth production (and FAs) to nearshore and deepwater foodwebs and commercially valuable fisheries.

## Supporting Information

Table S1 **Study sites and characteristics of 11 tributary systems of Lake Michigan.**
(DOCX)

Table S2 **Fatty acids measured in this analysis with reference materials.**
(DOCX)

Table S3 **Pearson correlation coefficients between total fatty acids and particular fatty acids or groups of fatty acids in seston, caddisflies and dreissenid mussels in tributary systems of Lake Michigan.**
(DOCX)

Appendix S1 **Geographic Appendix.** This is a file in the “keyhole markup language” (.kml) that can be viewed in “Google Maps” or “Google Earth”. This shows the specific locations where consumers and water was collected for this study (in much greater detail than Figure 2).
(DOC)

Appendix S2 **Data Appendix.** This file contains the raw data from the fatty acid analysis, the data used in this manuscript and copies of the datasets used in the statistical analyses described in the methods. Also included is metadata explaining variable names and individual sheets within the workbook.
(XLSX)

Appendix S3 **Expanded statistical methods.** This appendix provides an expanded description of the statistical analyses performed here, including example code (for use in R).
(DOCX)
