# Supplementary material for: Fatty Acid Composition at the Base of Aquatic Food Webs Is Influenced by Habitat Type and Watershed Land Use
Source: PLoS One. 2013 Aug 5;8(8):e70666. doi: 10.1371/journal.pone.0070666 (PMC3734252; doi:10.1371/journal.pone.0070666)
Supplement: Table S1 — Study sites and characteristics of 11 tributary systems of Lake Michigan. (DOCX) [file pone.0070666.s001.docx]

**Table S1.** Study sites and characteristics of 11 tributary systems of Lake Michigan.

|  |  |  |  | Watershed land cover (%) | |  |  |
| --- | --- | --- | --- | --- | --- | --- | --- |
| Site Name | Latitude | Longitude | Annual hydrograph (m^3^) | Surface water | Intense land use | Forest in buffer (%) | Distance from RM site to R site (km) |
| Kewaunee | 44° 27' 30" | 87° 33' 23" |  | 0.22 | 81.62 | 8.09 | 8.9 |
| Pike | 42° 38' 49" | 87° 51' 38" |  | 0.46 | 79.64 | 22.25 | 12.1 |
| Root | 42° 45' 05" | 87° 49' 25" |  | 0.62 | 79.27 | 6.73 | 7.4 |
| Manitowoc | 44° 06' 26" | 87° 42' 55" |  | 0.47 | 77.34 | 4.26 | 8.9 |
| Sheboygan | 43° 44' 29" | 87° 45' 08" |  | 1.55 | 73.83 | 3.92 | 5.7 |
| Oconto | 44° 51' 38" | 87° 59' 02" |  | 2.15 | 26.08 | 2.80 | 14.8 |
| Peshtigo | 45° 02' 51" | 87° 44' 40" |  | 3.21 | 19.02 | 5.21 | 12.0 |
| Pere Marquette | 43° 56' 42" | 86° 16' 43" |  | 1.25 | 17.64 | 15.89 | 16.2 |
| Betsie | 44° 33' 26" | 86° 03' 12" | NA | 9.97 | 16.20 | 24.55 | 20.0 |
| Ford | 45° 45' 18" | 87° 12' 07" |  | 0.30 | 6.27 | 28.68 | 14.8 |
| Sturgeon | 45° 56' 35" | 86° 42' 20" |  | 2.75 | 2.68 | 28.19 | 14.0 |
| Land cover (in percentage) is presented for the watershed upstream of the rivermouth. The distance from the RM to the R is the distance the river travels during average flow, not the straight-line distance. Hydrographs show the 10-year average (2001-2010) for each day from January 1st - December 31st (Note change in scale for Root and Pike hydrographs). Land cover characteristics are defined in the methods. More detailed location information is in the Geographic Appendix. | | | | | | | |
