# Supplementary material for: Fatty Acid Composition at the Base of Aquatic Food Webs Is Influenced by Habitat Type and Watershed Land Use
Source: PLoS One. 2013 Aug 5;8(8):e70666. doi: 10.1371/journal.pone.0070666 (PMC3734252; doi:10.1371/journal.pone.0070666)
Supplement: Table S2 — Fatty acids measured in this analysis with reference materials. (DOCX) [file pone.0070666.s002.docx]

**Table S2.** Fatty acids (FA) measured in this analysis. All FA measurements were estimated using a 5-point calibration curve except for those indicated by an asterisk (*), which were estimated using a 1-point calibration curve. The FA measured using a 1-point curve never made up more than 5% of the total FA. All of these FA were included in total FA (∑FA). PUFA- polyunsaturated fatty acids; MUFA- monounsaturated fatty acids.

| Fatty acid | Variables | Standard |
| --- | --- | --- |
| 14:0 |  | Supelco 47885-U |
| 14:1ω5 | MUFA | Supelco 47885-U |
| 15:0 |  | Supelco 47885-U |
| 15:1ω5 | MUFA | Supelco 47885-U |
| 16:0 |  | Supelco 47885-U |
| 16:1ω7 | MUFA | Supelco 47885-U |
| 17:0 |  | Supelco 47885-U |
| 18:0 |  | Supelco 47885-U |
| 18:1ω7 | MUFA | Nu-Chek Prep U-48-M |
| 18:1ω9c | MUFA | Supelco 47885-U |
| 18:1ω9t | MUFA | Supelco 47885-U |
| 18:2ω6c | ω6, PUFA, LIN | Supelco 47885-U |
| 18:2ω6t |  | Supelco 47885-U |
| 18:3ω3 | ω3, PUFA, ALA | Supelco 47885-U |
| 18:3ω6 | ω6, PUFA | Supelco 47885-U |
| 19:0 |  | Nu-Chek Prep U-19-M |
| 20:0 |  | Supelco 47885-U |
| 20:1ω9 | MUFA | Supelco 47885-U |
| 20:2ω6 | ω6, PUFA | Supelco 47885-U |
| 20:3ω3 | ω3, PUFA | Supelco 47885-U |
| 20:3ω6 | ω6, PUFA | Supelco 47885-U |
| 20:3ω9 | PUFA | Matreya 1179 |
| 20:4ω6 | ω6, PUFA | Supelco 47885-U |
| 20:5ω3 | ω3, PUFA, EPA | Supelco 47885-U |
| 21:0 |  | Supelco 47885-U |
| 22:0 |  | Supelco 47885-U |
| 22:1ω9 | MUFA | Supelco 47885-U |
| 22:2ω6 | ω6, PUFA | Supelco 47885-U |
| 22:4ω6 | ω6, PUFA, ARA | Nu-Chek Prep U-83-M |
| 22:5ω3 | ω3, PUFA | Supelco 47563-U |
| 22:5ω6 | ω6, PUFA | Nu-Chek Prep U-102-M |
| 22:6ω3 | ω3, PUFA, DHA | Supelco 47885-U |
| 23:0 |  | Supelco 47885-U |
| 24:0 |  | Supelco 47885-U |
| 24:1ω9 | MUFA | Supelco 47885-U |
| 26:0 |  | Matreya 1252 |
| 15:0*anteiso* |  | Matreya 1114* |
| 15:0*iso* |  | Matreya 1114* |
| 16:0*iso* |  | Matreya 1114* |
| *cis*-9,10-methylenehexadecanoate |  | Matreya 1114* |
| 17:0*iso* |  | Matreya 1114* |
| *cis*-9,10-methyleneoctadecanoate |  | Matreya 1114* |
