# Supplementary material for: Fatty Acid Composition at the Base of Aquatic Food Webs Is Influenced by Habitat Type and Watershed Land Use
Source: PLoS One. 2013 Aug 5;8(8):e70666. doi: 10.1371/journal.pone.0070666 (PMC3734252; doi:10.1371/journal.pone.0070666)
Supplement: Table S3 — Pearson correlation coefficients between total fatty acids and particular fatty acids or groups of fatty acids in seston, caddisflies and dreissenid mussels in tributary systems of Lake Michigan. (DOCX) [file pone.0070666.s003.docx]

**Table S3**. Pearson correlation coefficients between total fatty acids (∑FA μg L^-1^) and particular fatty acids (FA) or groups of FAs in seston, caddisflies (CF) and dreissenid mussels (DM) in tributary systems of Lake Michigan (USA).

| Variable | Units | PUFA | MUFA | EPA | DHA | ARA | ALA | LIN |
| --- | --- | --- | --- | --- | --- | --- | --- | --- |

| ∑FA Seston | μg L^-1^ | 0.99 | 0.97 | 0.95 | 0.97 | 0.94 | 0.96 | 0.91 |
| --- | --- | --- | --- | --- | --- | --- | --- | --- |
|  | μg mg^-1^ TSS | 0.99 | 0.99 | 0.86 | 0.88 | 0.91 | 0.98 | 0.97 |
| ∑FA CF | μg mg^-1^ DW | 0.96 | 0.98 | 0.25 | 0.87 | 0.82 | 0.81 | 0.63 |
| ∑FA DM | μg mg^-1^ DW | 0.98 | 0.98 | 0.97 | 0.91 | -0.36 | 0.79 | 0.80 |

| PUFA - polyunsaturated FA; MUFA - monounsaturated FA; EPA - 20:5ω3, eicosapentaneoic acid; DHA - 22:6ω3, docosahexaenoic acid; ARA - 20:4ω6, arachidonic acid; ALA - 18:3ω3 α-linolenic acid; LIN - 18:2ω6, linoleic acid. |
| --- |
