# Supplementary material for: Fatty Acid Composition at the Base of Aquatic Food Webs Is Influenced by Habitat Type and Watershed Land Use
Source: PLoS One. 2013 Aug 5;8(8):e70666. doi: 10.1371/journal.pone.0070666 (PMC3734252; doi:10.1371/journal.pone.0070666)
Supplement: Appendix S3 — Expanded statistical methods. This appendix provides an expanded description of the statistical analyses performed here, including example code (for use in R). (DOCX) [file pone.0070666.s006.docx]

**S3. Expanded statistical methods**

Statistical analysis was conducted in R version 2.11.1 [1], using the BRugs package, version 0.7-5 [2]. Essentially, this allows R to interface with the OpenBUGS program to run Markov chain Monte Carlo simulations of models [3,4].

Means and 95% credible intervals for seston and consumers at each site type (R, RM and L) were estimated following the methodology of McCarthy [4]. The following code is an example that was used to estimate the mean and 95% credible interval of seston data:

library("BRugs") ## this package allows R to talk with OpenBUGS

RSes <- read.csv("C:/Users/jhlarson/Desktop/USGS Science/Landscape Lipid Paper/Analysis/Means/RSes.csv") ## This is the data file

attach(RSes) ## This allows R to see the columns as variables

## The following is the function built in a format OpenBUGS can read

regressionmodel <- function(){

for (i in 1:11){

TSS[i]~dnorm(TSSmu[1],TSStau[1]) ##We used normal distributions

chla[i]~dnorm(chlamu[1],chlatau[1]) ## mu= mean, tau= precision

O3O6[i]~dnorm(O3O6mu[1],O3O6tau[1])

TFA[i]~dnorm(TFAmu[1],TFAtau[1])

PUFA[i]~dnorm(PUFAmu[1],PUFAtau[1])

MUFA[i]~dnorm(MUFAmu[1],MUFAtau[1])

EPA[i]~dnorm(EPAmu[1],EPAtau[1])

DHA[i]~dnorm(DHAmu[1],DHAtau[1])

ARA[i]~dnorm(ARAmu[1],ARAtau[1])

ALA[i]~dnorm(ALAmu[1],ALAtau[1])

LIN[i]~dnorm(LINmu[1],LINtau[1])

TFAM[i]~dnorm(TFAMmu[1],TFAMtau[1])

PUFAM[i]~dnorm(PUFAMmu[1],PUFAMtau[1])

MUFAM[i]~dnorm(MUFAMmu[1],MUFAMtau[1])

EPAM[i]~dnorm(EPAMmu[1],EPAMtau[1])

DHAM[i]~dnorm(DHAMmu[1],DHAMtau[1])

ARAM[i]~dnorm(ARAMmu[1],ARAMtau[1])

ALAM[i]~dnorm(ALAMmu[1],ALAMtau[1])

LINM[i]~dnorm(LINMmu[1],LINMtau[1])

}

TSSmu[1]~dnorm(0,0.001) ##Prior distributions were all uninformative

chlamu[1]~dnorm(0,0.001)## These are priors for the means

O3O6mu[1]~dnorm(0,0.001)

TFAmu[1]~dnorm(0,0.001)

PUFAmu[1]~dnorm(0,0.001)

MUFAmu[1]~dnorm(0,0.001)

EPAmu[1]~dnorm(0,0.001)

DHAmu[1]~dnorm(0,0.001)

ARAmu[1]~dnorm(0,0.001)

ALAmu[1]~dnorm(0,0.001)

LINmu[1]~dnorm(0,0.001)

TFAMmu[1]~dnorm(0,0.001)

PUFAMmu[1]~dnorm(0,0.001)

MUFAMmu[1]~dnorm(0,0.001)

EPAMmu[1]~dnorm(0,0.001)

DHAMmu[1]~dnorm(0,0.001)

ARAMmu[1]~dnorm(0,0.001)

ALAMmu[1]~dnorm(0,0.001)

LINMmu[1]~dnorm(0,0.001)

TSStau[1]~dgamma(0.001,0.001) ## Priors for precision

chlatau[1]~dgamma(0.001,0.001)

O3O6tau[1]~dgamma(0.001,0.001)

TFAtau[1]~dgamma(0.001,0.001)

PUFAtau[1]~dgamma(0.001,0.001)

MUFAtau[1]~dgamma(0.001,0.001)

EPAtau[1]~dgamma(0.001,0.001)

DHAtau[1]~dgamma(0.001,0.001)

ARAtau[1]~dgamma(0.001,0.001)

ALAtau[1]~dgamma(0.001,0.001)

LINtau[1]~dgamma(0.001,0.001)

TFAMtau[1]~dgamma(0.001,0.001)

PUFAMtau[1]~dgamma(0.001,0.001)

MUFAMtau[1]~dgamma(0.001,0.001)

EPAMtau[1]~dgamma(0.001,0.001)

DHAMtau[1]~dgamma(0.001,0.001)

ARAMtau[1]~dgamma(0.001,0.001)

ALAMtau[1]~dgamma(0.001,0.001)

LINMtau[1]~dgamma(0.001,0.001)

}

regressionmodelfile <- file.path(tempdir(),"regressionmodel.txt")

model <- writeModel(regressionmodel,regressionmodelfile)##Prep model

inits <- "C:\\Users\\jhlarson\\Desktop\\USGS Science\\OpenBugs Code\\18meansinitial.txt"

##Initials were stored in a text file (reproduced in the next line)

##list(TSSmu=c(0),chlamu=c(0),O3O6mu=c(0),TFAmu=c(0),PUFAmu=c(0),MUFAmu=c(0),EPAmu=c(0),DHAmu=c(0),ARAmu=c(0),ALAmu=c(0),LINmu=c(0),TFAMmu=c(0),PUFAMmu=c(0),MUFAMmu=c(0),EPAMmu=c(0),DHAMmu=c(0),ARAMmu=c(0),ALAMmu=c(0),LINMmu=c(0),TSStau=c(1),chlatau=c(1),O3O6tau=c(1),TFAtau=c(1),PUFAtau=c(1),MUFAtau=c(1),EPAtau=c(1),DHAtau=c(1),ARAtau=c(1),ALAtau=c(1),LINtau=c(1),TFAMtau=c(1),PUFAMtau=c(1),MUFAMtau=c(1),EPAMtau=c(1),DHAMtau=c(1),ARAMtau=c(1),ALAMtau=c(1),LINMtau=c(1))

## Puts the data into OpenBUGS format

bdata <-bugsData(c("TSS","chla","O3O6","TFA","PUFA","MUFA","EPA","DHA","ARA","ALA","LIN","TFAM","PUFAM","MUFAM","EPAM","DHAM","ARAM","ALAM","LINM"),,digits=5)

modelCheck(regressionmodelfile) ##Load the model

modelData(bdata) ## Load the data

modelCompile(numChains=1) ## Compile the model

modelInits(inits,) ## Initialize the model

modelUpdate(50000) ## 50,000 updates as 'burn-in'

samplesSet(c("TSSmu","chlamu","O3O6mu","TFAmu","PUFAmu","MUFAmu","EPAmu","DHAmu","ARAmu","ALAmu","LINmu","TFAMmu","PUFAMmu","MUFAMmu","EPAMmu","DHAMmu","ARAMmu","ALAMmu","LINMmu")) ## Tells OpenBUGS to watch these distributions

modelUpdate(50000) ## 50,000 updates for sampling

RSesMeans<- samplesStats("*") ##Reports the distributions

## This process now repeats using RM and L data

RMSes <- read.csv("C:/Users/jhlarson/Desktop/USGS Science/Landscape Lipid Paper/Analysis/Means/RMSes.csv")

attach(RMSes)

bdata <-bugsData(c("TSS","chla","O3O6","TFA","PUFA","MUFA","EPA","DHA","ARA","ALA","LIN","TFAM","PUFAM","MUFAM","EPAM","DHAM","ARAM","ALAM","LINM"),,digits=5)

modelCheck(regressionmodelfile)

modelData(bdata)

modelCompile(numChains=1)

modelInits(inits,)

modelUpdate(50000)

samplesSet(c("TSSmu","chlamu","O3O6mu","TFAmu","PUFAmu","MUFAmu","EPAmu","DHAmu","ARAmu","ALAmu","LINmu","TFAMmu","PUFAMmu","MUFAMmu","EPAMmu","DHAMmu","ARAMmu","ALAMmu","LINMmu"))

modelUpdate(50000)

RMSesMeans<- samplesStats("*")

LSes <- read.csv("C:/Users/jhlarson/Desktop/USGS Science/Landscape Lipid Paper/Analysis/Means/LSes.csv")

attach(LSes)

bdata <-bugsData(c("TSS","chla","O3O6","TFA","PUFA","MUFA","EPA","DHA","ARA","ALA","LIN","TFAM","PUFAM","MUFAM","EPAM","DHAM","ARAM","ALAM","LINM"),,digits=5)

modelCheck(regressionmodelfile)

modelData(bdata)

modelCompile(numChains=1)

modelInits(inits,)

modelUpdate(50000)

samplesSet(c("TSSmu","chlamu","O3O6mu","TFAmu","PUFAmu","MUFAmu","EPAmu","DHAmu","ARAmu","ALAmu","LINmu","TFAMmu","PUFAMmu","MUFAMmu","EPAMmu","DHAMmu","ARAMmu","ALAMmu","LINMmu"))

modelUpdate(50000)

LSesMeans<- samplesStats("*")

## End of example

For the regression models used in this analysis, the data was standardized by subtracting the mean from each value, then dividing by the standard deviation [5]. A detailed example of the code used for the regression is provided below. This example was used for estimating regressions of the dreissenid mussel fatty acid composition in the nearshore of Lake Michigan. The .csv file used in this example is included as a worksheet in the Data Appendix. Other than changing the variables as necessary, this code was also used on seston from each ecosystem type (river, rivermouth, nearshore zone), river caddisflies and rivermouth dreissenid mussels (see Methods).

Example code (with explanation) follows. In R, text following "#" symbol is not part of the code, and so explanations have been embedded in the code following the "##" symbol. File locations have been left in as examples, but for others repeating the analysis, these file locations need to be altered to the appropriate location (or the data must be imported some other way). Variable names are explained in the Data Appendix.

######

library("BRugs")

## This package allows R to communicate with OpenBUGS

## Importing the data

LZMStd <- read.csv("C:/Users/jhlarson/Desktop/USGS Science/Landscape Lipid Paper/Analysis/StandardizedSlopes/LZMStd.csv")

attach(LZMStd) ## This allows R to see columns as variables

## Standardized regression between FA property x and land cover y

regressionmodel <- function(){

a~dnorm(0,1.0E-6)

## Intercept, normal distribution with non-informative prior

b~dnorm(0,1.0E-6) ## Slope, normal distribution with non-informative prior

prec~dgamma(0.001,0.001)

## Error in regression, gamma distribution with non-informative prior

for (i in 1:10)

{

mean [i] <- a+b*x[i] ## This is the regression equation

y[i] ~dnorm(mean[i],prec)

}

}

##This model is then written in a form that OpenBUGS can read.

regressionmodelfile <- file.path(tempdir(),"regressionmodel.txt")

model <- writeModel(regressionmodel,regressionmodelfile)

inits <- "C:\\Users\\jhlarson\\Desktop\\USGS Science\\OpenBugs Code\\Regression\\Initials.txt"

##location of initial file; initials = list(a=0, b=0, prec=100)

x <- Water ## Assigning the predictor variable as x

## Assigning the variables as x and y allows the same model to be used in all the following analysis

##Slopes for Water

y <- TFAM

bdata <-bugsData(c("y","x"),,digits=5)

modelCheck(regressionmodelfile)

modelData(bdata)

modelCompile(numChains=1)

modelInits(inits,)

modelUpdate(50000)

samplesSet(c("b"))

modelUpdate(50000)

SlopeTFAM <- samplesStats("*")

## This is then repeated for all the FA response variables, plus TSS and Chl a for seston

y <- EPAM

bdata <-bugsData(c("y","x"),,digits=5)

modelCheck(regressionmodelfile)

modelData(bdata)

modelCompile(numChains=1)

modelInits(inits,)

modelUpdate(50000)

samplesSet(c("b"))

modelUpdate(50000)

SlopeEPAM <- samplesStats("*")

y <- ARAM

bdata <-bugsData(c("y","x"),,digits=5)

modelCheck(regressionmodelfile)

modelData(bdata)

modelCompile(numChains=1)

modelInits(inits,)

modelUpdate(50000)

samplesSet(c("b"))

modelUpdate(50000)

SlopeARAM <- samplesStats("*")

y <- O3O6

bdata <-bugsData(c("y","x"),,digits=5)

modelCheck(regressionmodelfile)

modelData(bdata)

modelCompile(numChains=1)

modelInits(inits,)

modelUpdate(50000)

samplesSet(c("b"))

modelUpdate(50000)

SlopeO3O6 <- samplesStats("*")

## This function binds together all those individual slope estimates

LConWater <- rbind(SlopeO3O6,SlopeTFAM,SlopeEPAM, SlopeARAM)

## The whole process is then repeated for the next predictor variable

x <- Dist

y <- TFAM

bdata <-bugsData(c("y","x"),,digits=5)

modelCheck(regressionmodelfile)

modelData(bdata)

modelCompile(numChains=1)

modelInits(inits,)

modelUpdate(50000)

samplesSet(c("b"))

modelUpdate(50000)

SlopeTFAM <- samplesStats("*")

y <- O3O6

bdata <-bugsData(c("y","x"),,digits=5)

modelCheck(regressionmodelfile)

modelData(bdata)

modelCompile(numChains=1)

modelInits(inits,)

modelUpdate(50000)

samplesSet(c("b"))

modelUpdate(50000)

SlopeO3O6 <- samplesStats("*")

y <- EPAM

bdata <-bugsData(c("y","x"),,digits=5)

modelCheck(regressionmodelfile)

modelData(bdata)

modelCompile(numChains=1)

modelInits(inits,)

modelUpdate(50000)

samplesSet(c("b"))

modelUpdate(50000)

SlopeEPAM <- samplesStats("*")

y <- ARAM

bdata <-bugsData(c("y","x"),,digits=5)

modelCheck(regressionmodelfile)

modelData(bdata)

modelCompile(numChains=1)

modelInits(inits,)

modelUpdate(50000)

samplesSet(c("b"))

modelUpdate(50000)

SlopeARAM <- samplesStats("*")

LConDist <- rbind(SlopeO3O6,SlopeTFAM,SlopeEPAM, SlopeARAM)

x <- ForBuff

y <- O3O6

bdata <-bugsData(c("y","x"),,digits=5)

modelCheck(regressionmodelfile)

modelData(bdata)

modelCompile(numChains=1)

modelInits(inits,)

modelUpdate(50000)

samplesSet(c("b"))

modelUpdate(50000)

SlopeO3O6 <- samplesStats("*")

y <- TFAM

bdata <-bugsData(c("y","x"),,digits=5)

modelCheck(regressionmodelfile)

modelData(bdata)

modelCompile(numChains=1)

modelInits(inits,)

modelUpdate(50000)

samplesSet(c("b"))

modelUpdate(50000)

SlopeTFAM <- samplesStats("*")

y <- EPAM

bdata <-bugsData(c("y","x"),,digits=5)

modelCheck(regressionmodelfile)

modelData(bdata)

modelCompile(numChains=1)

modelInits(inits,)

modelUpdate(50000)

samplesSet(c("b"))

modelUpdate(50000)

SlopeEPAM <- samplesStats("*")

y <- ARAM

bdata <-bugsData(c("y","x"),,digits=5)

modelCheck(regressionmodelfile)

modelData(bdata)

modelCompile(numChains=1)

modelInits(inits,)

modelUpdate(50000)

samplesSet(c("b"))

modelUpdate(50000)

SlopeARAM <- samplesStats("*")

LConForBuff <- rbind(SlopeO3O6,SlopeTFAM,SlopeEPAM, SlopeARAM)

## End of example

Literature Cited:

1. R Development Core Team (2010) R: A language and environment for statistical computing.

2. Thomas A, O’Hara B, Ligges U, Sturtz S (2006) Making BUGS open. R News 6: 12–16.

3. Carlin BP, Louis TA (2008) Bayesian methods for data analysis. 3rd ed. Boca Raton, FL: Chapman & Hall/CRC. p.

4. McCarthy M (2007) Bayesian methods for ecology. New York, New York, USA: Cambridge University Press. p.

5. Hair JF, Anderson RE, Tatham RL, Black WC (1998) Multivariate data analysis. 5th ed. Upper Saddle River, New Jersey, USA: Prentic-Hall, Inc. p.
